# Supplementary material for: Ni-Catalyzed Enantioselective Intramolecular Mizoroki–Heck Reaction for the Synthesis of Phenanthridinone Derivatives
Source: J Org Chem. 2023 Jun 15;88(13):8203–26. doi: 10.1021/acs.joc.3c00202 (PMC10337041; doi:10.1021/acs.joc.3c00202)
Supplement: Supplementary file 2 — jo3c00202_si_002.zip [file jo3c00202_si_002.zip › Amides(N-H and N-Me)/S2i-I iPr-NH-4F-ArI/19F iPr-NH-4F-ArI S2i-I/1/pdata/1/email_YK-114-081 F6 CDCl3 F19_1_1.pdf]

YK-114-081 F6 CDCl3 F19
F19 CDCl3 {D:\nmrusers\malachowski} BM 16

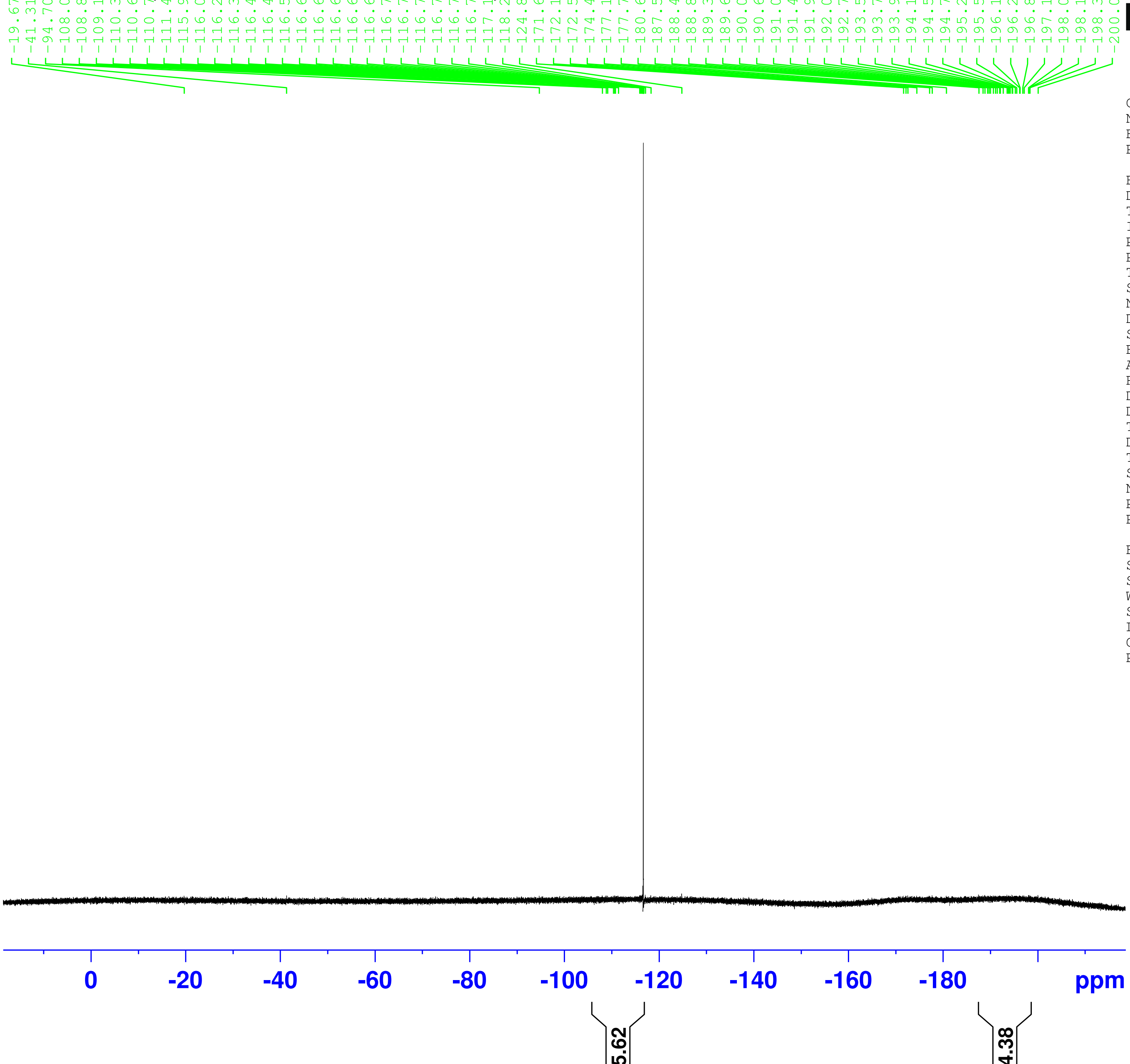

BRUKER

Current Data Parameters

NAME	YK-114-081 F6 CDCl3 F19
EXPNO	1
PROCNO	1

F2 - Acquisition Parameters

Date_	20220707
Time	12.09 h
INSTRUM	spect
PROBHD	Z104450_0352 (
PULPROG	zgflqn
TD	131072
SOLVENT	CDCl3
NS	16
DS	4
SWH	89285.711 Hz
FIDRES	1.362392 Hz
AQ	0.7340032 sec
RG	203
DW	5.600 usec
DE	6.50 usec
TE	298.1 K
D1	1.00000000 sec
TD0	1
SFO1	376.4795333 MHz
NUC1	19F
P1	12.30 usec
PLW1	19.19499969 W

F2 - Processing parameters

SI	65536
SF	376.5171850 MHz
WDW	EM
SSB	0
LB	0.30 Hz
GB	0
PC	1.00
